# The Effect of a DNA Repair Gene on Cellular Invasiveness: Xrcc3 Over-Expression in Breast Cancer Cells

**DOI:** 10.1371/journal.pone.0016394

**Published:** 2011-01-24

**Authors:** Veronica L. Martinez-Marignac, Amélie Rodrigue, David Davidson, Martin Couillard, Ala-Eddin Al-Moustafa, Mark Abramovitz, William D. Foulkes, Jean-Yves Masson, Raquel Aloyz

**Affiliations:** 1 McGill University, Lady Davis Institute & Segal Cancer Center, Jewish General Hospital, Montreal, Canada; 2 Faculty of Medicine, Program in Cancer Genetics, McGill University, Montreal, Canada; 3 Department of Oncology, McGill University, Montreal, Canada; 4 Genome Stability Laboratory, Laval University Cancer Research Center, Hôtel-Dieu de Québec, Québec City, Canada; Health Canada, Canada

## Abstract

Over-expression of DNA repair genes has been associated with resistance to radiation and DNA-damage induced by chemotherapeutic agents such as cisplatin. More recently, based on the analysis of genome expression profiling, it was proposed that over-expression of DNA repair genes enhances the invasive behaviour of tumour cells. In this study we present experimental evidence utilizing functional assays to test this hypothesis. We assessed the effect of the DNA repair proteins known as X-ray complementing protein 3 (XRCC3) and RAD51, to the invasive behavior of the MCF-7 luminal epithelial-like and BT20 basal-like triple negative human breast cancer cell lines. We report that stable or transient over-expression of XRCC3 but not RAD51 increased invasiveness in both cell lines *in vitro*. Moreover, XRCC3 over-expressing MCF-7 cells also showed a higher tumorigenesis *in vivo* and this phenotype was associated with increased activity of the metalloproteinase MMP-9 and the expression of known modulators of cell-cell adhesion and metastasis such as CD44, ID-1, DDR1 and TFF1. Our results suggest that in addition to its' role in facilitating repair of DNA damage, XRCC3 affects invasiveness of breast cancer cell lines and the expression of genes associated with cell adhesion and invasion.

## Introduction

Breast cancer is the leading cause of tumour-related death among women worldwide [1]. In Canada, breast cancer is the second cause for mortality after lung cancer and is the most frequently diagnosed cancer in women with a 2% increase of new diagnosed cases in respect to the 2009 statistics [2].

Currently therapeutic approaches are limited by the development of drug resistance and progression of the majority of tumours to a more invasive and aggressive phenotype [3]. Resistance to anticancer agents that induce DNA damage has been associated with increased expression of DNA repair genes [4], [5], [6], [7] and the development of aggressive/metastatic behaviour in at least four different types of tumours [8], [9], [10], [11], [12], [13], [14]. Recently, using gene expression profiling of human primary malignant melanoma, Sarasin and Kauffman [12] hypothesised that aberrant expression of genes connected with DNA repair pathways is associated with increased metastatic potential. In particular, over-expression of genes involved in double-strand break (DSB) repair and surveillance of DNA replication forks were associated with an increased tendency to generate highly malignant metastatic cancer cells and poor patient survival prognosis [9], [11], [14]. Although this concept has been hypothesized repeatedly there remains little experimental evidence to support it [12], [15].

The two major DNA repair pathways involved in the repair of DSB are the DNA homologous recombinational repair pathway (HRR) and the nonhomologous DNA end-joining (NHEJ) pathway. A functional link between a NHEJ DNA repair component and phenotypic changes in human cancer cells have been made. It has been shown that a component of the NHEJ repair complex, the protein KU80, is involved in cell-cell and cell-matrix adhesion [16], [17]. In addition to its central role in DNA DSB repair, KU80 co-localizes with metalloproteinase 9 (MMP-9) on the outer cell membrane and plays an important role in regulating extracellular matrix remodelling in highly invasive hematopoietic cells [16], [18]. MMP-9 mediates invasion of mammary carcinoma cells and invasion/angiogenesis of keratocyte tumours by binding to the hyaluronan receptor, CD44 [19], [20]. The reported association of the KU80/MMP-9 complex with the invasiveness of cancer cells is the first evidence linking DNA repair proteins with cellular invasiveness.

XRCC3 is a RAD51 paralog that participates in the HRR pathway [5], [21], [22], [23]. RAD51 catalyzes the invasion of an undamaged DNA template during homologous recombination, a crucial step leading to repair of a broken DNA. RAD51 localization to DSBs depends on the function and its direct interaction with XRCC3 [24]. XRCC3 has been reported to interact as well with BRCA2, FANCD2 and FANCG to form a discrete complex related to HRR and chromosome stability [24], [25]. It is known that XRCC3 expression levels are associated with increased DNA repair and resistance to the DNA damaging agents such as cisplatin and melphalan [7], [26], [27].

As the roles of XRCC3 and RAD51 on cellular invasiveness are unknown, we explored the phenotypic effect resulting in the over-expression of XRCC3 and RAD51 on different breast cancer cell lines, vis-^-vis invasiveness. Specifically, we over-expressed XRCC3 in two breast cancer cell lines, MCF-7 and BT20, with contrasting phenotypes and clinical prognosis [28]. MCF-7 is a luminal epithelial-like cell line which is positive for HER2, oestrogen and progesterone receptors expression while BT20 is a triple negative basal-like cell line, negative for HER2, oestrogen and progesterone receptor expression. Though both cell lines have contrasting phenotypes and clinical prognosis [28] they showed an increase in their invasiveness behaviour that was dependent of XRCC3 over-expression. The following study is the first report that test the hypothesis that over-expression of DNA repair genes enhances the invasive behaviour of tumour cells [9], [11], [12], [15].

## Materials and Methods

### Breast cancer cell lines

The breast cancer cell lines, MCF-7 and BT20 were purchased from American Type Culture Collection (ATCC) and cultivated in RPMI 1640 medium supplemented with 10% fetal bovine serum (FBS) (Gibco) in a humidified incubator maintained at 37°C and 5% CO_2_. Stably transfected pCDNA3.0-XRCC3 over-expressing and pCDNA3.0 MOCK MCF-7 cell lines produced in a previous study were used to investigate the effects of XRCC3 over-expression on the tumorigenesis and metastasis of breast cancer cells [7]. We also used transiently transfected XRCC3 and RAD51 MCF-7 and BT20 cells, the parental cell lines were obtained from American Type Culture Collection (ATCC). In this case, MCF-7 and BT20 cells were transfected with pCDNA3.0 or pCDNA3.0 carrying the XRCC3 cDNA or RAD51 cDNA using Superfect or Lipofectamine 2000 according to the manufacturer's directions. Briefly, cells were plated at a density of 2×10^6^ cells/plate on 100 mm tissue culture dishes. After overnight incubation, the transfection mix containing 30 µg of plasmid DNA was applied. Cells were incubated for an additional 48 hrs and harvested. Over-expression of XRCC3 and RAD51 protein in stably or transiently transfected MCF-7 and BT20 cells was confirmed by Western blot analysis.

### Western Blotting

Basal expression level of proteins related with DNA repair and cell-matrix adhesion were characterised by Western blots. Aliquots (100 µg protein) of cell lysate were loaded onto 4-12% gradient acrylamide gels. After each electrophoresis and transfer, the following antibodies were used: from Cell Signalling, MMP-9 that recognizes the pro enzyme (92 kDa) and the cleaved active form (84 kDa); from Santa Cruz Biotechnology, Inc, anti-ID-1 (C-20), DDR1 (C-20), RAD51 (sc8349) and anti-Actin (I-19); from Oncogen, we used anti-XRCC3 (Oncogen, San Diego, CA. Ab-1); from Neomarkers KU70 (Ab4- MS 329- P0) and KU80 (Ab7- MS 332-P0). Goat anti-Actin (Santa Cruz Biotechnology, Inc) or mouse monoclonal antibody against GAPDH (Research Diagnostics) was used to confirm protein loading and the blots were visualized using peroxidase substrate system (ECL Western blotting detection reagents, Millipore).

### Matrigel invasion assays

Matrigel-coated invasion chamber plates of 8.0-µm pore size (BD Bioscientific) were used for the invasion assay. Cells (5×10^4^ cells/0.5 mL) were plated in the top chamber in FBS-free RPMI 1640 media and culture medium with 10% FBS RPMI 1640 was used in the bottom chamber as a chemo-attractant. Forty-eight hours later, cells were fixed using a 3.7% formaldehyde solution and stained using Hematoxylin-EosinY (Thermo Scientific). Cell numbers were counted over four fields of the porous membrane and photographed. As in previous reports, MDA MB-436 cell line was used as a positive control [29]. The invasion assay was replicated on stably transfected MOCK and XRCC3 OE MCF-7 and on MOCK, XRCC3 and RAD51 OE transiently transfected MCF-7 and BT20 cells.

### Gene silencing of XRCC3 gene by small interference RNA

RNAi-mediated knockdown of XRCC3 was conducted using a siRNA (Dharmacon) with CAGAATTATTGCTGCAATTAA as a target sequence. As negative control, a non-silencing siRNA (Dharmacon) with target sequence CGTCATATACCAAGCTAGTT was used. Transfection was performed using Oligofectamine (Invitrogen), according to the manufacturer's protocol with minor modifications. We selected the MCF-7 over-expressing cell as it was previously reported that the silencing of Xrcc3 gene showed to reduce the proliferation of MCF-7 cells by the accumulation of DNA breaks [21]. In brief, stably XRCC3 over-expressing MCF7 cells were seeded in six-well plates at 2×10^5^ cells/well the day prior to transfection. For each transfection, the cells were exposed to 250 nM of siRNA in serum- and antibiotic-free Opti-MEM (Invitrogen) during 4 hours and then supplemented with 500 µl of RPMI 1640 containing 30% FBS. After 18 hrs, cells (15×10^4^ cells/0.5 mL) were plated in the top chamber of a Matrigel-coated invasion plate (BD Bioscientific) and the invasion capacity of the cells was assessed as described above.

### Zymogram: MMP-9

To assess the presence of pro-MMP-9 and MMP-2 and active MMP-9 and MMP-2, serum-free, 24 hr-conditioned media was harvested from sub-confluent cultures of 3×10^6^ cells. Volumes representing equivalent cell numbers were concentrated 10 times by Amicon centrifugation using a 10 kDa cut-off and 30 µl of concentrated medium was then separated on 10% SDS-PAGE containing 0.1% gelatin pre-casted gels (BioRAD). Gels were incubated in PBS containing 2.5% Triton X-100 at room temperature for 1 h, followed by further incubation in substrate-collagenase buffer. Gelatinolytic activity was visualized following 0.5% Coomassie Blue G-250 staining and destaining with 30% methanol, 10% acetic acid solution. Gels were digitally photographed and band densitometry performed using Scion image software (Scion Corporation, Frederick, MA, USA). Equivalent loading was confirmed by Coomassie staining of a gel run in parallel and the presence of MMP9 was verified on a parallel gel treated with 25 mM EDTA as inhibitor of MMP-9 activity.

### RNA isolation and RT-PCR (reverse transcription-polymerase chain reaction)

Total RNA was extracted from MOCK and XRCC3 OE MCF-7 cells using the RNeasy kit (Qiagen cat#74104). Seven different cell cultures were used with the number of passages ranging from 4 to 17. The purity and quantification of all RNA preparations were monitored using NanoDrop ND-1000 spectrophotometer.

#### TFF1 or Sp2

RNA samples were reverse transcribed using the QuantiTect reverse transcription kit (Qiagen cat#205313) and expression was analyzed by quantitative real time PCR using primers as follows: TFF1, forward 5′-accatggagacaaggtgat-3′ and reverse 5′-aaattcacactcctcttctg-3′; and GAPDH as an internal control, forward 5′-tgcaccaccaactgcttagc-3′ and reverse 5′-ggcatggactgtggtcatgag-3′. All reactions were performed in triplicate in a Sybr Green PCR master mix (ABI cat#4309155) containing 0.25 µM of each primer. Conditions for amplification were 10 min at 95°C followed by 40 cycles at 95°C, 15 s; and 60°C, 60 s; in a 7500 real time PCR system (ABI). A melting curve analysis was included to monitor PCR specificity.

#### CD44

CD44 mRNA expression was analyzed by semi-quantitative One-Step RT-PCR assay (Qiagen cat# 210210) using 0.25 µM of forward primer 5′gacacatattgcttcaatgcttcagc 3′ and a reverse primer 5′-gatgccaagatgatcagccattctggaa-3′ [30]. The reactions were performed in triplicate and the conditions for reverse-transcription and PCR were 1 cycle of 30 minutes at 55°C followed by a denaturation step of 2 minutes at 94°C, and 35 cycles at 94°C, 15 s; 55°C, 20 s; and 68°C, 30 s. This reaction results in the amplification of a 482 base-pair fragment that spans the region between exon 3 and 4 which was visualized by a 2% agarose gel.

### Flow cytometry analysis

A flow cytometric (FCM) analysis was performed to assess expression of the membrane form of CD44 and membrane and cellular forms of KU80. We used a FACS-Calibur instrument (Becton Dickinson) and Cell-Quest software. PE-conjugated mouse anti-human CD44 (BD-Pharmingen™ PE 555479) antibody was used. An isotype-matched antibody (PE-conjugated rabbit, BD) was used as a control. For KU80, after fixation with 1% paraformaldehyde at 4°C for 15 minutes, cells were washed with PBS then, in the case of total KU80 determination they were permeabilized in 70% cold ethanol for 20 minutes in ice. After washing in PBS, the cells were incubated 30 minutes at room temperature in dilution buffer (PBS, 1% BSA, 0.1% triton X100) and then, incubated overnight at 4°C with a mouse anti-KU80 antibody (Ab7- MS 332-P0, Neomarkers). The cells were washed in PBS, and then incubated for 90 minutes with a goat anti-mouse Alexa Fluor 488 secondary antibody (Molecular Probe, Eugene, OR, USA) diluted 1∶500 in dilution buffer, washed with PBS, finally analyzed by FCM as previously described.

### Cell fractionation: Nuclear and cytoplasmic extracts

Nuclear and cytoplasmic cell fractionation was performed using approximately 5×10^6^ cells homogenized in 200 µl of buffer A (10 mM HEPES at pH 7.9, 50 mM NaCl, 500 mM sucrose, 0.15 mM spermine, 0.5 mM spermidine, 1.0 mM EDTA, 0.2% TX-100, mercaptoethanol 7 mM and mini protease inhibitor cocktail–tablet, Roche). Nuclei were spun out in a refrigerated microfuge. The supernatant (cytoplasmic extract) was kept for further use and the pellet was washed in 500 µl of buffer B (as A with 25% glycerol and without sucrose and TX-100) and then suspended in 50 µl of buffer C (350 mM NaCl, 10 mM HEPES pH 7.9, 25% glycerol, 0.15 mM spermine, 0.5 mM spermidine, 1.0 mM EDTA), then centrifuged at 4°C and the supernatant was stored as nuclear extract.

We assessed expression of XRCC3 and KU80 proteins on 60% confluent cultures by Western blot of nuclear and cytoplasmic fractions. Rabbit anti-XRCC3 (Oncogen, San Diego, CA. Ab-1) was used and mouse anti-p53 (Neomarkers, Ab8) and Rho A (Santa Cruz Biotechnology, Inc) were used to confirm nuclear and cytoplasmic fractions.

### *In vivo* studies, mouse xenograft model

Stably transfected MOCK and XRCC3 OE MCF-7 cells were grown to 70% confluence, and were harvested from the subconfluent cultures by exposure to 0.25% trypsin and 0.02% EDTA. Trypsinization was stopped by the addition of medium containing 10% FBS. Cells were kept at 4°C until use (0–1 hour). Cell concentration and viability (Trypan blue exclusion, 90% viability) were determined using a hemocytometer. Seven and eight C.B17 SCID female mice (Fox Chase Scid-Charles River) were injected subcutaneously into the flanks with 1.5×10^6^ exponentially growing MOCK and XRCC3 OE MCF-7 cells, respectively, in 100 µl of sterile PBS, pH 7.4. The female mice of 10 weeks old were used for xenograft studies in accordance with the guidelines of the Canadian Council on Animal Care and with appropriate institutional certification (Animal Welfare Assurance No.A5006-01, IACUC status approved -11/01/2009- McGill University Animal Care Committee and Jewish General Hospital, Montreal, Canada).

Tumour growth was confined to local masses and did not affect animal survival over a 6-month observation period. Tumour volumes were monitored weekly by caliper measurement of the length and width, and their volumes calculated according to the modified ellipsoidal formula (V =  (l x w^2^)/2) [31]. The mice were sacrificed and tumours were collected and fixed in paraffin when the tumours reached more than 100 mm^3^.

### Immunohistochemistry

Immunoperoxidase staining for PCNA and XRCC3 was performed by the avidin-biotin complex method (Vector Laboratories, Burlingame, CA). 4-µm formalin fixed paraffin embedded sections were cut, placed on SuperFrost/Plus slides (Fisher), and dried overnight at 37°C. Sections were deparaffinised in xylene and rehydrated through graded alcohols to water; then they were immersed in 10 mM sodium citrate buffer, pH 6.0, and subjected to heat-induced antigen retrieval. Endogenous peroxidase activity was quenched by incubation in 3% hydrogen peroxide for 15 min. Endogenous biotin was blocked by incubation for 10 min with the Avidin/Biotin blocking kit (Zymed Laboratories Inc., San Francisco, CA). To block non specific protein binding, sections were then treated with 5% normal goat serum in PBS-Tween 0.1% (PBS-T) for 60 min at room temperature. Sections were incubated overnight at 4°C with primary antibodies at appropriate dilutions made in blocking solution: mouse anti-PCNA (Novocastra) at 1∶100 and rabbit anti-XRCC3 1∶25 (Abcam, Inc.). After rinsing with PBS-T, sections were incubated with biotinylated antibodies (goat anti- mouse or goat anti-rabbit, Vector Laboratories) for 60 min at room temperature. The sections were then incubated for 30 min with avidin-biotin-horseradish peroxidase complex (Vector Laboratories), followed by final color development with peroxidase substrate kit DAB (Vector Laboratories) for 3–5 min. A negative control was performed by the omission of the primary antibody. Sections were then lightly counterstained with hematoxylin, dehydrated in graded alcohols, cleared in xylene and coverslipped. Sections were analyzed by conventional light microscopy.

### Statistical and image analysis

Statistical analyses were performed using Sigma Stat and PlotStat packages. The analysis included the Mann-Whitney U test and Student's unpaired and paired *t* test (for two groups). Comparison among group tumour incidence was performed by a Kaplan Meier analysis and plot and the probability of a trend in the incidence score through a logrank test. Data are expressed as fold differences between means and were considered significant at *P*<0.05. For image analysis ImageJ 1.41o or Scion Corporation software was used.

## Results

### Changes in the expression of XRCC3 but not RAD51 modulate the invasiveness of human breast cancer cells *in vitro*

**Figure 1:**
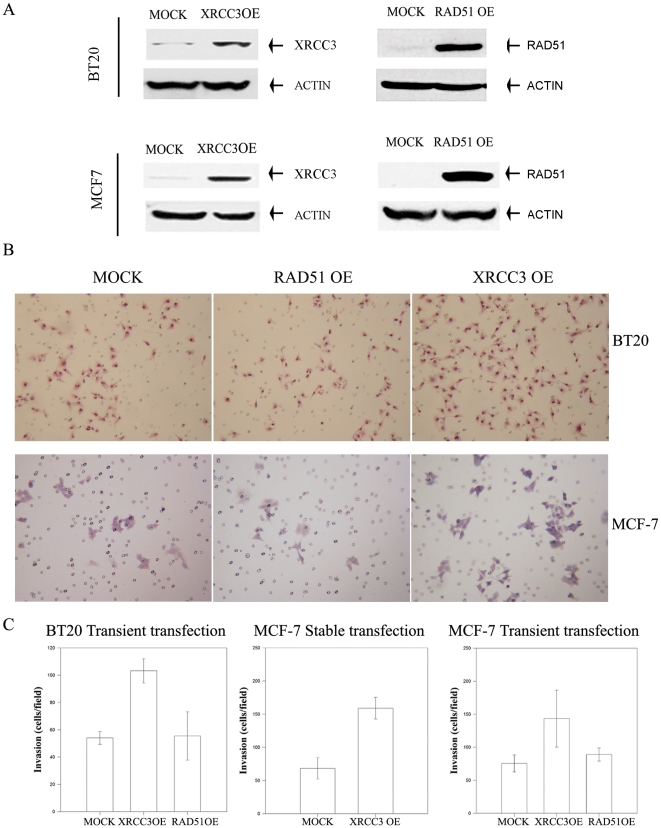
Assessing the invasion phenotype of XRCC3 over-expressing cells. (A) Independent XRCC3 and RAD51 transient transfections were assayed and their protein expression was assessed through Western Blot. (B) MOCK and RAD51 or XRCC3 OE BT20 and MCF-7 cells were seeded onto Matrigel covering the surface of a porous membrane. Cells were incubated for 48 hrs to allow penetration of the matrix. Invading cells were subsequently fixed using 3.7% formaldehyde solution and stained using Hematoxylin-EosinY (Thermo Scientific). Migrating/invading cells were counted. (C) Data was then plotted as mean cells per field ±SD for a minimum of three separate experiments, each in duplicate. XRCC3 OE cells had a much higher degree of basal motility and invasion than did their MOCK BT20 and MCF-7 counterparts (2 fold higher, P<0.01) while RAD51 OE cells were not significantly different from MOCK cells.

XRCC3 and RAD51 over-expression was assessed by Western blot. RAD51 and XRCC3 transient transfected MCF-7 and BT20 cells over-expressed RAD51 or XRCC3 by 2- to more than 10-fold, when compared with MOCK-transfected cells (Fig. 1A). MCF-7 stably transfected with XRCC3 overexpress XRCC3 by more than 3-fold, when compared with the stably transfected MOCK cells (Table 1). Using a Boyden chamber assay, a classical method to assess invasiveness also named trans-well migration assay [17], [32], [33], [34], we found that XRCC3 over-expressing MCF-7 and BT20 cells (XRCC3 OE hereafter) were two-fold more invasive than their mock transfected counterparts (MOCK hereafter) (Fig. 1B–C). These increases in invasiveness were statistically significant with p values of <0.01 and 0.002 for MCF-7 cells and BT20 cells respectively. The higher invasive phenotype was exclusively observed on XRCC3 OE cells and not on cells over-expressing RAD51 (RAD51 OE hereafter) and the increase in invasiveness was dependent on the level of expression of XRCC3 (Figure S1). The increase in XRCC3 OE cells invasiveness and its dependency on XRCC3 protein expression levels was also confirmed through gene silencing of the Xrcc3 gene by small interfering RNA in MCF-7 cells stably over-expressing XRCC3 (Figure S2).

**Table 1:** Expression of biomarkers associated with metastasis or invasion analyzed by different methods in MCF-7 cells.

MARKER	Fold differences	MOCK	XRCC3 OE	P value	METHOD
CD44 protein	2.6	22%±7	57%±12	0.041	FCM*
CD44 mRNA	2.5	0.4±0.05	1.0±0.03	<0.001	RT-PCR, Semi-quantitativeπ
DDR1 protein	3.8	0.4±0.16	1.6±0.43	0.029	Western blot¥
ID-1 protein	9.0	0.1±0.06	1.2±0.48	0.043	Western blot
KU70 total protein	1.0	1.1±0.25	1.1±0.26	0.979	Western blot
KU80 total protein	1.0	0.8±0.21	0.9±0.01	1.000	Western blot
KU80, membrane associated form	1.3	34%±7	41%±4	0.333	FCM
MMP-9 protein	4.5	0.4±0.18	1.8±0.48	0.033	Western blot
Pro-MMP-9 protein	3.4	28±3.96	96±3.62	<0.001	Zymogram
Active-MMP-9 protein	2.0	42±7.77	87±13.8	0.045	Zymogram
XRCC3 protein	3.0	0.4±0.15	1.3±0.25	0.024	Western blot
XRCC3 -OE 1 protein	10.0	0.1	1.0	N.A.	Western blot
XRCC3 -OE 2 protein	20.0	0.1	2.0	N.A.	Western blot
TFF1/pS2 mRNA	2.4	Relative expression to MOCK		0.001	RT-PCR Quantitative

* % of cells expressing CD44 or KU80 on the outer membrane by flow cytometry analysis;

**π** values are relative to GAPDH mRNA expression;

**¥** values are relative to Actin expression.

### XRCC3 over-expression affects the expression of biomarkers associated with metastasis in human breast cancer cells

**Figure 2:**
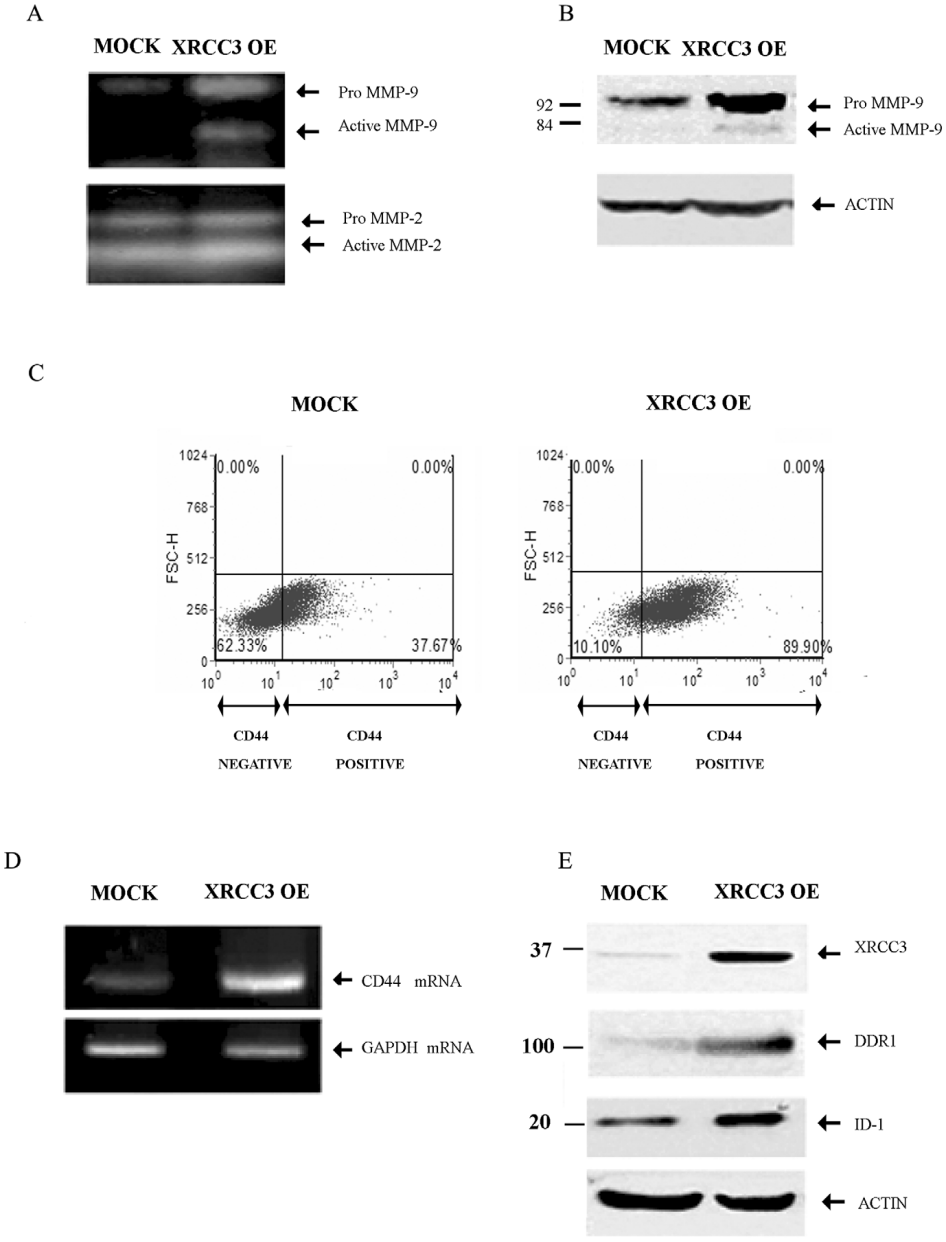
Characterization of the expression of proteins associated with ECM metabolism and invasion. (A) Zymography shows MMP-2 and MMP-9 activity in serum free MCF-7 conditioned medium. The gelanolytic bands of 84 (active) and 92 kDa (pro-active) MMP-9 were increased two- and more than three-fold, respectively (P = 0.045 and P<0.001), in XRCC3 OE cells with respect to MOCK cells (P<0.01); while there were no differences for MMP-2 bands. (B) MMP-9 protein levels were increased 4-fold in XRCC3 OE cells compared to MOCK cells (P = 0.033). (C) Using flow cytometry, we found that XRCC3 OE cells expressed significantly higher levels of CD44 protein than MOCK cells (two-fold, P = 0.041). (D) Using semi-quantitative reverse transcription PCR a two-fold increase in CD44 mRNA expression was observed in XRCC3 OE cells (P<0.001). (E) ID-1 and DDR1 protein levels were significantly increased (P = 0.043 and P = 0.029, respectively) in XRCC3 OE cells with respect to MOCK cells.

We analyzed MMP-2 and MMP-9 enzymatic activity in MCF-7 conditioned media by gelatin zymography. As shown in Fig. 2A, the gelatinolytic bands of 84 and 92 kDa of MMP-9 were increased more than two- and four-fold, respectively, in the culture supernatant of XRCC3 OE cells with respect to MOCK cells (P<0.01). In contrast, the 72-kDa gelatinolytic band, representing the product of the MMP-2 gene was unchanged by XRCC3 expression level (Fig. 2A). We also confirmed a significantly increased level of MMP-9 protein in XRCC3 OE MCF-7 cells lysates by Western blot analysis (Table 1). MMP-9 protein levels were up four-fold in XRCC3 OE cells with respect to MOCK cells (P = 0.03) (Fig. 2B, Table 1).

In contrast, to the proteins mentioned above, two other proteins KU80 (XRCC5) and KU70 (XRCC6) showed no significant differences between MOCK and XRCC3 OE MCF-7 cells with respect to the expression or cellular localization when analyzed by flow cytometry and Western blot analysis of cell fractions (Table 1).

In addition, using flow cytometry we found that XRCC3 OE MCF-7 cells expressed significantly higher levels of CD44 protein than MOCK cells as represented by the number of CD44 positive cells in the flow cytometric analysis (2-fold, P = 0.041) (Fig. 2C). A similar result was obtained using semi-quantitative reverse transcription PCR were a 2-fold increase in CD44 mRNA expression was observed in XRCC3 OE cells (Fig. 2D, Table 1).

Using the QuantiTect reverse transcription kit (Qiagen cat#205313) and quantitative real time PCR we also found that in XRCC3 OE cells, TFF1 mRNA level was two-fold higher in XRCC3 OE MCF-7 cells compared to MOCK cells (P<0.001) (Table 1).

We found by Western blot that ID-1 and DDR1 protein levels were also significantly increased in cell lysates, more than 3-fold (P = 0.043 and P = 0.029, respectively) in XRCC3 OE cells with respect to MOCK cells (Fig. 2E, Table 1).

### XRCC3 over-expression increase the tumorigenic capacity of MCF-7 cells *in vivo*

**Figure 3:**
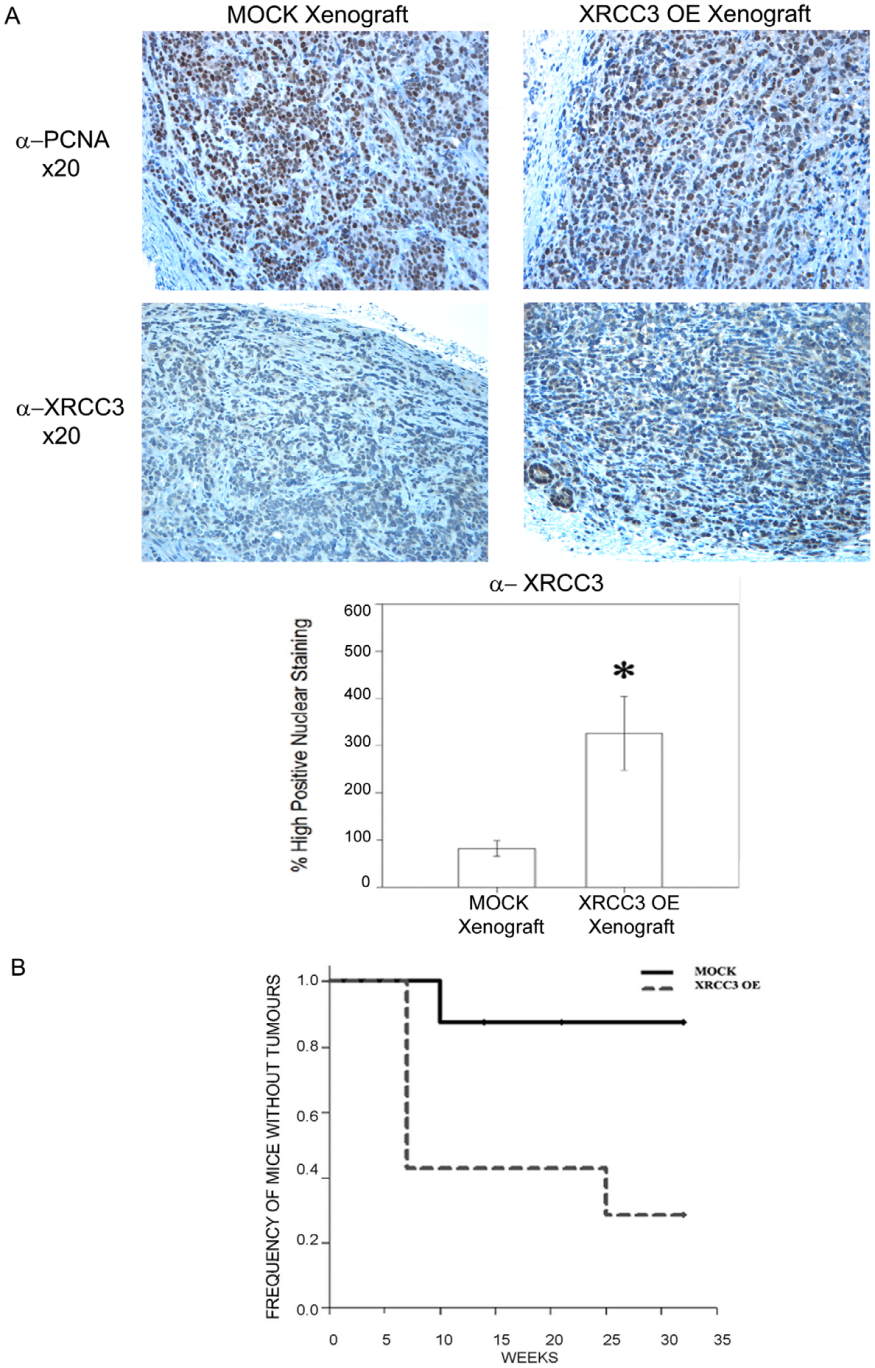
XRCC3 over-expression and MCF-7 xenograft growth in SCID mice. (A) Representative photomicrographs (×20) of anti-PCNA (Novocastra) and anti-XRCC3 (Abcam, Inc.) IHC performed on XRCC3 OE and MOCK consecutive tumour sections (*Left*) and anti-XRCC3 expression histograms (*Right*). The bar graphs represent the percentage of cells with high intensity nuclear staining for anti-XRCC3 (mean +/- standard deviation). The expression of PCNA showed no significant differences between the two tumour sections, while XRCC3 expression were significantly higher in XRCC3 OE tumours (P = 0.029), * significantly different P<0.05; (B) Kaplan Meier Survival plot showing that mice injected with XRCC3 OE cells tumours earlier and in a significantly higher incidence (P = 0.028) than the MOCK cells injected mice. After 12 weeks 4/7 and 1/8 mice showed tumours for XRCC3 OE and MOCK injections, respectively.

We subcutaneously injected SCID mice with equal numbers of MOCK or XRCC3 over-expressing MCF-7 cells (1.5×10^6^ cells) in the absence of oestradiol treatment. The tumour incidence was highly increased (P = 0.028) in mice injected with XRCC3 OE cells. After 12 weeks, 4/7 mice injected with XRCC3 OE showed tumours compared to 1/8 mice injected with MOCK cells (Fig. 3B). In addition, the xenograft analysis showed rapid tumour formation on those mice injected with XRCC3 OE cells. Mice injected with XRCC3 over-expressing cells developed tumours with volumes ranging from 1 to 8 mm^3^ at 4 weeks post injection. In contrast, at week ten post-injection we detected a 0.5 mm^3^ tumour in a mouse injected with MOCK cells (data not shown).

The mice were sacrificed when tumours exceeded 100 mm^3^ and tumours were collected and fixed in paraffin. The analysis of immunohistochemistry stainings for PCNA and XRCC3 showed no significant differences for PCNA expression; in contrast the expression of XRCC3 was shown in the cytoplasm and nucleus and was four-fold higher in the periphery of the XRCC3 OE cell tumours where 61% of the cells showed high nuclear staining. In contrast only 14% of MOCK tumour cells showed high levels of nuclear XRCC3 (P = 0.029) (Fig. 3A).

## Discussion

Our results demonstrate that 2-3 fold increase in the expression of XRCC3 protein levels is sufficient to significantly increase the invasive behaviour of two human breast cancer cell lines (MCF-7 and BT20) *in vitro*. These findings suggest that the proposed role of XRCC3 in the modulation of invasiveness might not be exclusive of a breast cancer subtype since MCF-7 is a luminal epithelial-like cell while BT20 is a triple negative basal-like cell line. Importantly, the increased invasiveness associated with XRCC3 over-expression was observed in both, stable and transient XRCC3 over-expressing cells suggesting that the effect is not likely due to secondary phenotypic alterations associated with clonal selection. The specificity of the effect of XRCC3 expression in invasion is further supported by the following two observations reported in this manuscript: 1) the dose effect, higher XRCC3 over-expression displayed higher invasive capacity and 2) decreasing levels of XRCC3 from treatment with a specific Xrcc3 siRNA led to decreasing invasive capacity.

Consistently with the changes in the invasive behaviour of the cells, we found that increased expression of XRCC3 resulted in increased activity of MMP-9, a gelatinase involved in the extracellular matrix (ECM) degradation that is thought to play an important role in cancer metastasis [16], [18], [19], [20]. Moreover, we found that the XRCC3 over-expression resulted in increased expression of CD44, the Discoidin Domain Receptor 1 (DDR1) and ID-1. These three biomarkers have been associated with increased invasiveness of cancer cells and/or localization and modulation of MMP-9 function [16], [32], [35], [36], [37], [38], [39]. CD44 has been shown to anchor the proteolytically-active form of MMP-9 to the cell surface [19] while the constitutive expression of either the transcription factor ID-1 or the receptor DDR1 has been reported to increase the activity of MMP-9 and the invasiveness of human cancer cells (35–39). These results demonstrate that XRCC3 expression can alter the expression/activity of a network of proteins associated with ECM metabolism, one of the key steps involved in cell invasion. Furthermore, XRCC3 over-expression resulted in a significant increase in the expression of the human trefoil protein TFF1 a modulator of cell motility, another key step required for cell invasion and metastasis. It has been shown that TFF1 (also known as pS2) stimulates the movement of MCF-7 and other breast cancer cells and promotes their dissemination by preventing anoikis [40], [41], [42], [43]. MCF-7 breast cancer cells expressing a higher level of TFF1 have an increased capacity to spread by invading surrounding tissues thus conferring them with a more invasive/metastatic phenotype [40], [41], [42], [43].

Taken together with our *in vitro* results, we demonstrated that XRCC3 expression levels affect the invasiveness of breast cancer cells, the expression/activity of targets associated with the regulation of invasiveness (i.e. TFF1), the interaction of cancer cells with the extracellular matrix (i.e. ID-1, DDR-1 and CD44) and the extracellular matrix turnover (i.e. MMP-9).

In an attempt to support the *in vitro* results we assessed the effect of XRCC3 over-expression on the growth of MCF-7 tumour xenografts in SCID mice under suboptimal conditions (i.e. in the absence of oestradiol treatment). MCF-7 has been reported to grow tumours poorly without oestradiol treatment in SCID mice [44], [45]. Our results show that XRCC3 favours the growth of MCF-7 xenografts since the growth rate of tumours was roughly five times higher for XRCC3 over-expressing cells when compared to the control type (i.e. 57% vs. 12% respectively). Importantly, immnocytochemical analysis using a specific XRCC3 antibody confirmed the over-expression of the protein in the xenograft tumour tissues. XRCC3 expression was four-fold higher in the XRCC3 OE cell tumours. Furthermore, 61% of XRCC3 OE tumour cells showed high nucleus staining compared with 14% of MOCK tumour cells. Interestingly, similar to previous reports [46], the tumours showed not only nuclear but also cytoplasmic XRCC3 staining in both kinds of xenograft derived tissues.

Even though it has been shown that XRCC3 over-expression does not affect the doubling time of MCF-7 cells *in vitro* [7], the effect *in vivo* has not been tested. To discard a possible effect of XRCC3 over-expression on the doubling time of MCF-7 cells *in vivo*, we investigated the expression of the Proliferating Cell Nuclear Antigen, PCNA. PCNA is an auxiliary protein of DNA polymerase whose level of synthesis correlates directly with rates of cellular proliferation and DNA synthesis; and it has been widely used as a marker to assess the mitotic index of tumour cells [47], [48]. In agreement with previous *in vitro* reports, XRCC3 over-expression did not affect the proliferative capability of MCF-7 cells, since there was no significant difference in PCNA expression between the MOCK and XRCC3 OE tumours.

Our results demonstrate that stable or transient over-expression of XRCC3 increases *in vitro* invasiveness in two breast cancer cell lines from different lineage (luminal and triple negative). Though we still lack a clear mechanism of how XRCC3, a protein associated with HRR, could affect the invasive phenotype of the cell lines we found that the phenotype and the over-expression were associated with increased activity of the metalloproteinase MMP-9 and the expression of known modulators of cell-cell adhesion and metastasis such as CD44, ID-1, DDR1 and TFF1. Moreover, XRCC3 over-expressing cells showed a higher tumorigenesis and higher XRCC3 expression *in vivo.*

XRCC3 plays an important role in both DNA repair and resistance to anticancer agents that induce DNA damage (6, 20–25). Our results indicate that over-expression of the XRCC3 gene is also associated with an invasive behaviour *in vitro* and the promotion of tumour formation *in vivo,* these features were independent of RAD51 and the proliferation rate of the cells. In addition, XRCC3 did not alter the expression levels of the membrane form of KU80, another DNA repair-related gene (designated XRCC5) that functions as a docking protein for MMP-9 at the cell surface [16], [17], [33]. These results taken together suggest that XRCC3 can affect MMP-9 activity independently of KU80 or KU70 (XRCC6).

In summary, the recently established XRCC3 over-expressing MCF-7 cell line [7] is a promising model for the study of drug resistance and malignant progression associated with DNA-repair pathways. This is the first report that provides experimental results showing that a DNA repair protein is involved in the regulation of invasiveness and tumour growth in human breast cancer cells and suggests that XRCC3 expression could be used as a biomarker for breast tumour growth and development.

## Supporting Information

Figure S1 **Invasiveness is dependent of XRCC3 expression level.** Two independent XRCC3 transient transfections were assayed on MCF-7 cells. XRCC3 OE cells showed a higher level of basal invasion than MOCK cells and the rate of invasion (A) corresponds to the level of XRCC3 protein over-expression detected by Western blot analysis (B) where XRCC3-OE 1 and XRCC3-OE 2 showed an increased expression of more than ten- and twenty-fold.
(TIF)

Figure S2 **XRCC3 siRNA of MCF-7 stably over-expressing XRCC3 reverts the phenotype to a lesser invasive one.** (**A**) Western blotting analysis of XRCC3 protein after transfection by XRCC3 siRNA of MCF-7 cells stably over-expressing XRCC3, XRCC3 protein was recognized by anti-*XRCC3* antibody (Oncogen). GAPDH (Research Diagnostics) was used as a loading control. XRCC3 protein expression of XRCC3 OE cells was reduced in a 23 to a 47%; (**B**) Invasion data for two separate experiments, each in triplicate. The siXRCC3 XRCC3 OE cells invasion relative to siControl showed less basal invasion than did their siControl-XRCC3 OE cells when the XRCC3 protein expression was reduced in 38 to 47% from the expression of their siControl-XRCC3 OE counterpart though to obtain a statistically significant effect on invasion XRCC3 has to be reduced more than the 40% of its protein expression.
(TIF)
