# Supplementary figures and images for: The Effect of a DNA Repair Gene on Cellular Invasiveness: Xrcc3 Over-Expression in Breast Cancer Cells
Source: PLoS One. 2011 Jan 24;6(1):e16394. doi: 10.1371/journal.pone.0016394 (PMC3025979; doi:10.1371/journal.pone.0016394)

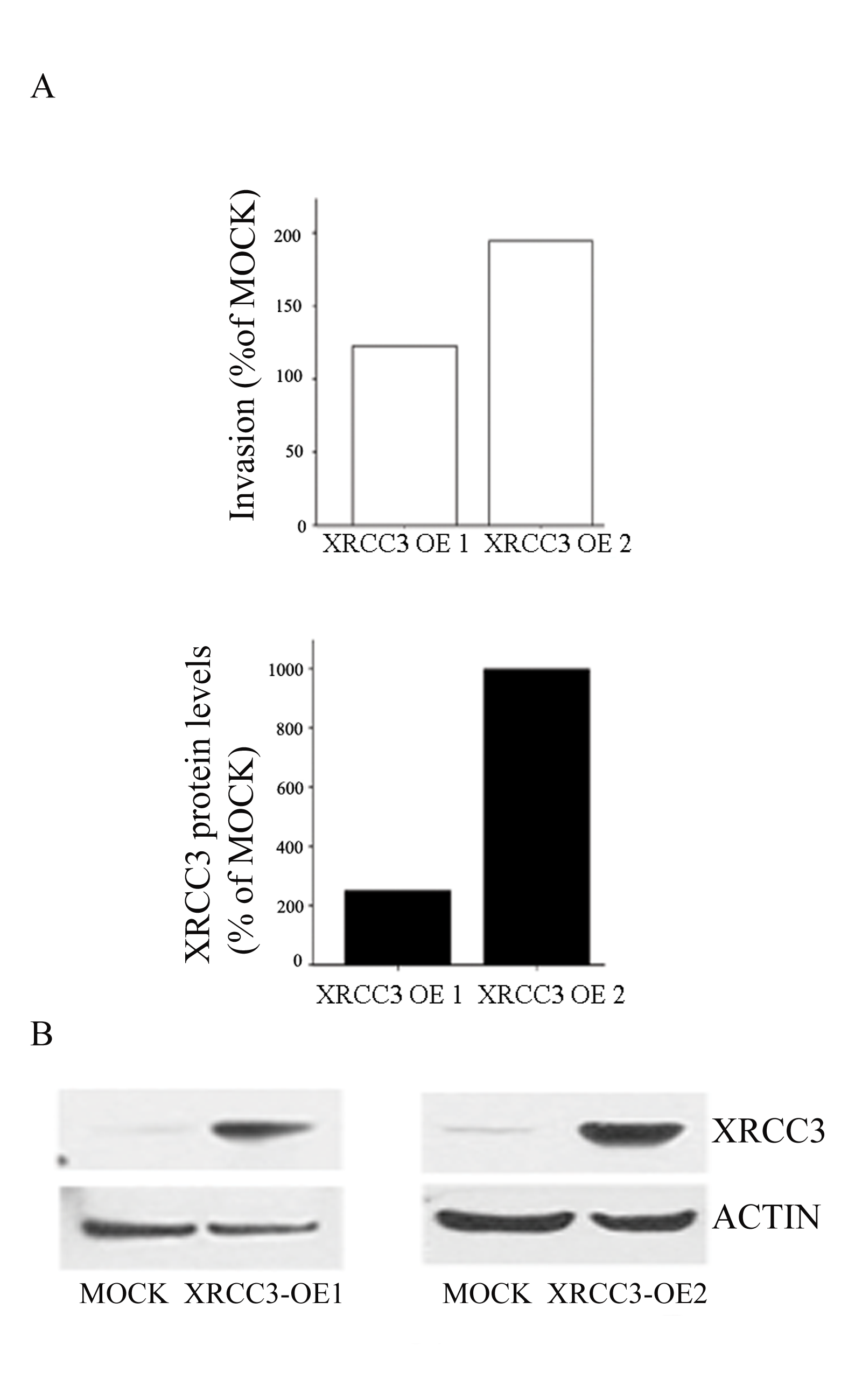

Supplement: Figure S1 — Invasiveness is dependent of XRCC3 expression level. Two independent XRCC3 transient transfections were assayed on MCF-7 cells. XRCC3 OE cells showed a higher level of basal invasion than MOCK cells and the rate of invasion (A) corresponds to the level of XRCC3 protein over-expression detected by Western blot analysis (B) where XRCC3-OE 1 and XRCC3-OE 2 showed an increased expression of more than ten- and twenty-fold. (TIF) [file pone.0016394.s001.tif]

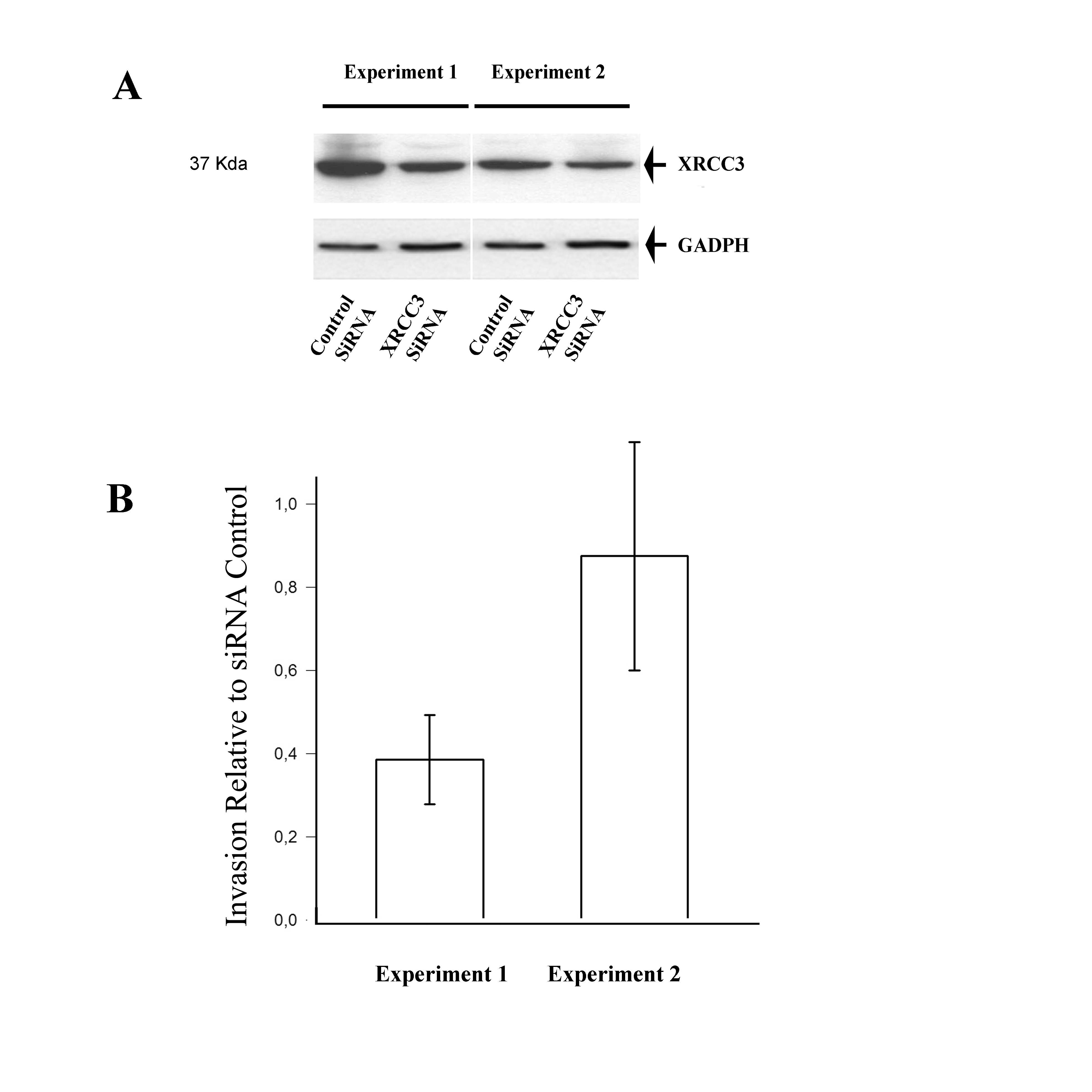

Supplement: Figure S2 — XRCC3 siRNA of MCF-7 stably over-expressing XRCC3 reverts the phenotype to a lesser invasive one. (A) Western blotting analysis of XRCC3 protein after transfection by XRCC3 siRNA of MCF-7 cells stably over-expressing XRCC3, XRCC3 protein was recognized by anti-XRCC3 antibody (Oncogen). GAPDH (Research Diagnostics) was used as a loading control. XRCC3 protein expression of XRCC3 OE cells was reduced in a 23 to a 47%; (B) Invasion data for two separate experiments, each in triplicate. The siXRCC3 XRCC3 OE cells invasion relative to siControl showed less basal invasion than did their siControl-XRCC3 OE cells when the XRCC3 protein expression was reduced in 38 to 47% from the expression of their siControl-XRCC3 OE counterpart though to obtain a statistically significant effect on invasion XRCC3 has to be reduced more than the 40% of its protein expression. (TIF) [file pone.0016394.s002.tif]
